# Editorial

**DOI:** 10.1120/jacmp.v7i3.2375

**Published:** 2006-08-24

We have several changes in the masthead of the JACMP for this summer issue. We welcome as new Associate Editors: Jefferson Fairbanks, William Salter and Albert Zacarias in Radiation Oncology Physics, and James Rodgers in Radiation Protection and Regulations. We also recognize the dedicated contributions of outgoing Associate Editors: Michael Herman and John Gibbons (Radiation Oncology Physics), James Deye (Radiation Protection & Regulations), and Richard Stark (Associate Editor at Large). Our heartfelt thanks go to the both of you for your years of dedicated service to the JACMP.

This quarter, it is my pleasure to welcome a guest editorial from Paul J. Early, DABSNM, DABR, DABNM, who is Corporate RSO with Digirad Corp. The issue he describes, validation of authorized users, will be of interest to every medical physicist that practices in the United States. In particular, if the medical physicist practices in a state that comes under the direct jurisdiction of the Nuclear Regulatory Commission, this issue may be of special concern. The neglect of information contained in this editorial could result in administrative penalties for the Licensee or the Authorized User.

Michael D. Mills, PhD

## Validation of Authorized Users

Paul J. Early, DABSNM, DABR, DABNM, Corporate RSO, Digirad Corp.

A recent NRC proceeding has established the NRC's official interpretation of certain regulations concerning duties the NRC believes are incumbent upon a licensee in verifying the accuracy of information provided by prospective Authorized Users (AUs) the licensee seeks to add to its license. These duties apply regardless of whether the prospective AUs are employees of the licensee, are independent contractors or are consulting organizations providing services.

## HISTORY

In the summer of 2004, the NRC Office of Investigations (OI) initiated an investigation to determine whether a physician (the “Physician”) listed on a licensee's (the “Licensee”) NRC radioactive material license (“RML”) had submitted false information to the Licensee to become an AU on the Licensee's existing NRC RML. Based on the evidence developed during its investigations, OI substantiated that the Physician had submitted false and/or inaccurate information to the Licensee for the purpose of being named as an AU on the Licensee's existing RML. Specifically, the NRC determined that the Physician had provided the Licensee with (a) a preceptor letter, signed by another AU, attesting that the Physician had received the required minimum level of supervised clinical and work experience to be an AU, and (b) a statement that the Physician was already an AU on another existing NRC license. Neither document was accurate.

Even though the NRC also determined that the Licensee had been unaware of the falseness or inaccuracy of the information, the NRC took the position that the Licensee's act of submitting that information to the NRC constituted a potential violation by the Licensee itself, and began enforcement proceedings against the Licensee. The Licensee and the NRC then agreed to participate in an alternative dispute resolution session (“ADR”) to resolve this apparent violation and pending enforcement action. ADR is a process in which a neutral mediator, with no decision‐making authority, assists the parties in resolving any disagreements on whether a violation occurred, agreeing on the appropriate enforcement action (if any), and stipulating to the appropriate corrective measures. That ADR session was held in late 2005.

## POSITION TAKEN BY THE PARTIES BEFORE THE MEDIATION

The NRC took the position that, in submitting the inaccurate information, the Licensee acted in “careless disregard of NRC requirements”. The contention of the Office of Enforcement was that, even though the Licensee was unaware of the inaccuracy of the information submitted, a violation had occurred because licensees are responsible for the acts and omissions of their agents. The Licensee disagreed with this interpretation of the regulations, and contended that, because it had not been aware of any inaccuracy, it had not committed a violation.

## CORRECTIVE ACTIONS BY THE LICENSEE PRIOR TO THE ADR SESSION

Subsequent to becoming aware of the NRC investigation and its results, and of the apparent violation, the Licensee took several actions to assure that these events would not recur. These actions included:

1. Immediately removing the Physician from its license
2. Canceling an existing business relationship with the Physician
3. Attaching to physician and preceptor statements the following notice:
*“Notice to Physician and Preceptor: 10 CFR 30.9(a) and 30.10(a) require that all information provided to the NRC by a licensee or its agents shall be complete and accurate in all material respects. The submission of false information constitutes a serious violation of applicable regulations and may cause you or us to be fined, to lose licensing privileges, or to suffer other significant penalties.”*
4. Requiring any physician added to its license to sign and date a document containing a statement equivalent to the following:
*“In connection with my application to be named as an Authorized User on [Licensee's] radioactive material license, I am aware that the submission of information that is not complete and accurate in all material respects is a violation of 10 CFR Sections 30.9(a) and 30.10(a). I hereby represent and warrant that, to the best of my knowledge, the information I have submitted to [Licensee] in connection with my application to be named as an Authorized User is complete and accurate in all material respects.”*

## RESULTS OF THE ADR SESSION

An ADR session was held between the NRC and the Licensee in the NRC Region I headquarters in King of Prussia, Pa in late 2005. As described above, the session was mediated by a professional mediator. As a result of that session, as well as subsequent discussions, a settlement was reached. The elements of the settlement agreement include the following:

1. The NRC and the Licensee agreed to disagree on the interpretation of the regulations as to whether the violation represented careless disregard of NRC requirements. The Licensee continued to maintain that its submission of inaccurate information was not in careless disregard of NRC requirements since it had no knowledge of the inaccuracies in the information provided to it by the AU.
2. The Licensee agreed that the Physician, listed as an AU on its RML, had provided inaccurate information to the Licensee to become an AU on its license.
3. The NRC agreed that the Licensee did not knowingly submit the inaccurate information to the NRC, but nonetheless, the NRC maintained that a violation in careless disregard of NRC requirements occurred because a licensee is responsible for the acts and omissions of its agents.
4. The Licensee agreed that it must submit complete and accurate information to the NRC in accordance with 10 CFR 30.9(a).

## ADDITIONAL CORRECTIVE ACTIONS BY THE LICENSEE FOLLOWING THE ADR SESSION

In addition to the corrective actions taken by the Licensee before the ADR session, the Licensee took the following additional corrective actions:

1. The Licensee agreed, for all future NRC AU applicants and on a yearly basis, to audit the training and experience credentials of the first 10 AU applicants and 25% of any applications received after the first 10. The audit will include an attempt to locate and call preceptors as well as CME providers to verify the information given by the AU applicants. The Licensee also agreed to submit the results of this audit to the NRC at the end of a two year period, as well as to notify the NRC immediately after identification of any discrepancies as a result of the audit. The parties agreed that, if no falsifications are uncovered during the two year period, the Licensee will discontinue the audit procedure.
2. An officer of the Licensee agreed to prepare and submit a commentary on this event to a variety of scientific journals addressing Nuclear Medicine and/or Medical Physics, as well as to include such commentary in future lectures, to provide an opportunity for other licensees in the industry to learn from this incident.
3. In light of the corrective actions the Licensee had already taken prior to the ADR and the further corrective actions agreed upon, the NRC agreed to issue a Severity Level III Notice of Violation, but not to impose a civil penalty.

## FOLLOW UP

An issue not covered in the ADR was the extent to which the Licensee could reasonably rely on information provided by third parties. This has particular significance when the evidence for satisfying AU credentials consists of (1) the prospective AU already being named as an AU on another NRC/Agreement State RML, or (2) the prospective AU having been certified by the Certification Board of Nuclear Cardiology (“CBNC”) or any other professional board recognized by the NRC. Can a Licensee be held responsible for any lack of due diligence on the part of these credentialing entities that supported an application in either of these situations? The Licensee thus directed a letter to the Office of General Counsel (“OGC”) of the NRC, posing the following question: “Can [the Licensee] rely, without the need for independent verification, on the fact that the physician seeking to be named on our license has already been named as an AU on another license (NRC and/or Agreement State) or has already been certified by one of the approved certification boards … as proof of the requisite training and experience?” The OGC responded that the answer to this question is, “yes”, provided a licensee:

1. Verifies that the board certifying the physician is one that is currently recognized by the NRC;
2. Verifies that the physician is listed as an AU on an NRC or Agreement State license; and
3. Verifies that no escalated enforcement action, which could jeopardize the standing of the applicant as a qualified individual, has been issued to the physician by either the NRC or an Agreement State by accessing: http://www.nrc.gov/what‐we‐do/regulatory/enforcement.html.

## SUMMARY

Since licensees are responsible for the acts as well as the omissions of its employees and agents, the NRC believes that the above steps of verification are necessary as applicable. This is true whether the prospective AU is applying to be named as an AU for the first time or has been named on another RML or is certified by a NRC‐recognized board.

**Table:** 

The Journal of Applied Clinical Medical Physics	
**Editorial Board 2006**	
Editor‐in‐Chief	Michael Mills
Deputy Editor‐in‐Chief	Timothy Solberg
**Associate Editors**	
Radiation Oncology Physics	Nzhde Agazaryan, Salahuddin Ahmad Jefferson Fairbanks, B. Gino Fallone Janelle Molloy, Matthew Podgorsak Nikos Papanikolaou, William Salter Mehrdad Sarfaraz, Lu Wang, Albert Zacarias
Medical Imaging	Geoffrey Clarke, William Pavlicek
Radiation Measurements	Larry DeWerd
Radiation Protection & Regulations	James Rodgers
Nonionizing Topics	Bhudatt Paliwal
Other Topics	Timothy Solberg
Book/Media Reviews	James Smathers
Editorials	John Horton
**Journal Production Team**	
Proofreader	Heather Shand
Copy Editor	Betty Robinson
Layout Editor	Laura Shand
Manuscript Manager	Patricia Walker

